# Supplementary material for: Hybrides of Alkaloid Lappaconitine with Pyrimidine Motif on the Anthranilic Acid Moiety: Design, Synthesis, and Investigation of Antinociceptive Potency
Source: Molecules. 2020 Nov 27;25(23):5578. doi: 10.3390/molecules25235578 (PMC7730767; doi:10.3390/molecules25235578)
Supplement: Supplementary file 1 [file molecules-25-05578-s001.pdf]

Molecules

Supporting Information

Optimization of alkaloid lappaconitine with pyrimidine motif on the anthranilic acid moiety: Design, synthesis and investigation of antinociceptive potency

Kirill P. Cheremnykh [1], Victor A. Savelyev [1], Sergey A. Borisov [1], Igor D. Ivanov [2], Dmitry S. Baev [1], Tatyana G. Tolstikova [1], Valentin A. Vavilin [2], Elvira E. Shults [1*]

[1] *N.N. Vorozhtsov Novosibirsk Institute of Organic Chemistry, Siberian Branch of the Russian Academy of Sciences, Lavrentyev Ave, 9, 630090 Novosibirsk, Russian Federation*

[2] *The Federal Research Center Insitute of Molecular Biology and Biophysics, 2/12, Timakov St., Novosibirsk, 630117, Russia.*

Content:

^{1}H and ^{13}C NMR spectra of final compounds……………………………S2-S13

✉ Elvira E. Shults

schultz@nioch.nsc.ru

[1] Laboratory of Medicinal Chemistry, Novosibirsk Institute of Organic Chemistry, Siberian Branch of the Russian Academy of Sciences, Lavrentyev Ave, 9, 630090 Novosibirsk, Russia;

12

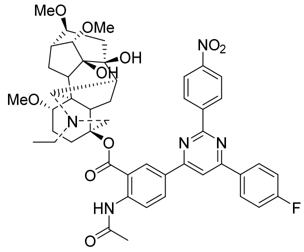

^{1}H NMR ($CDCl_3$, 400 MHz)

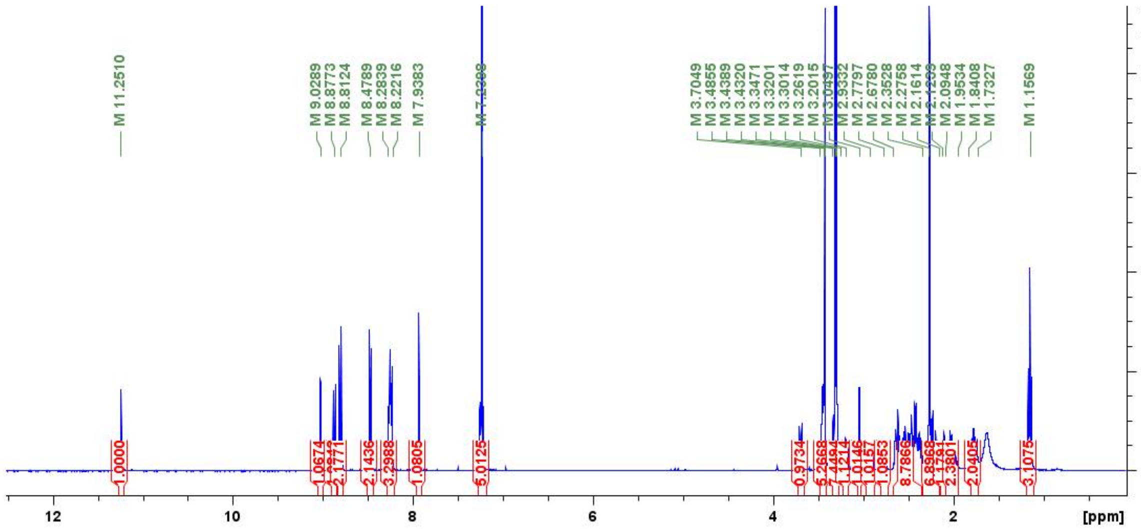

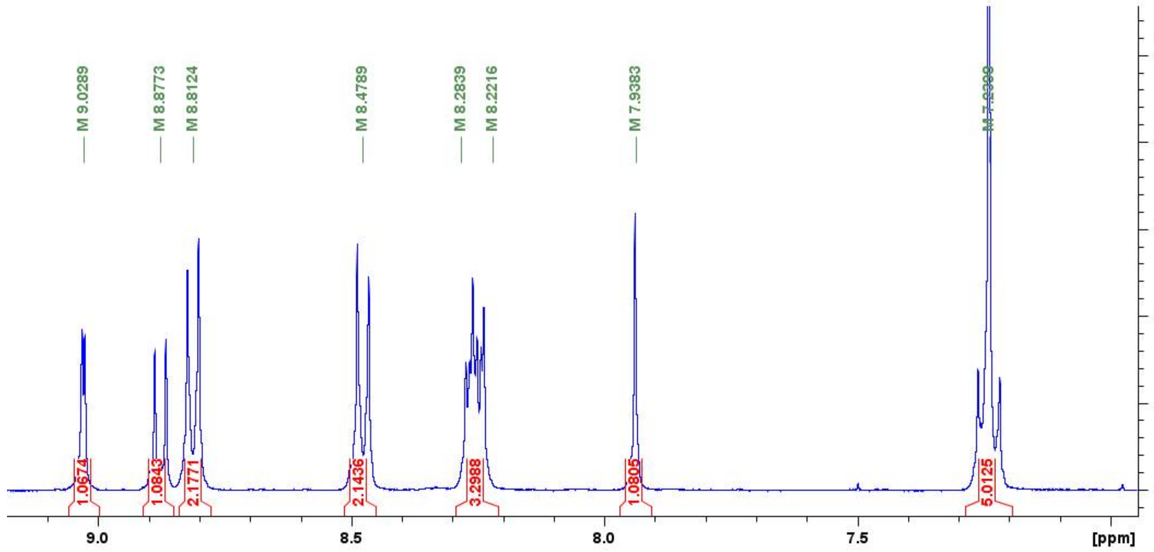

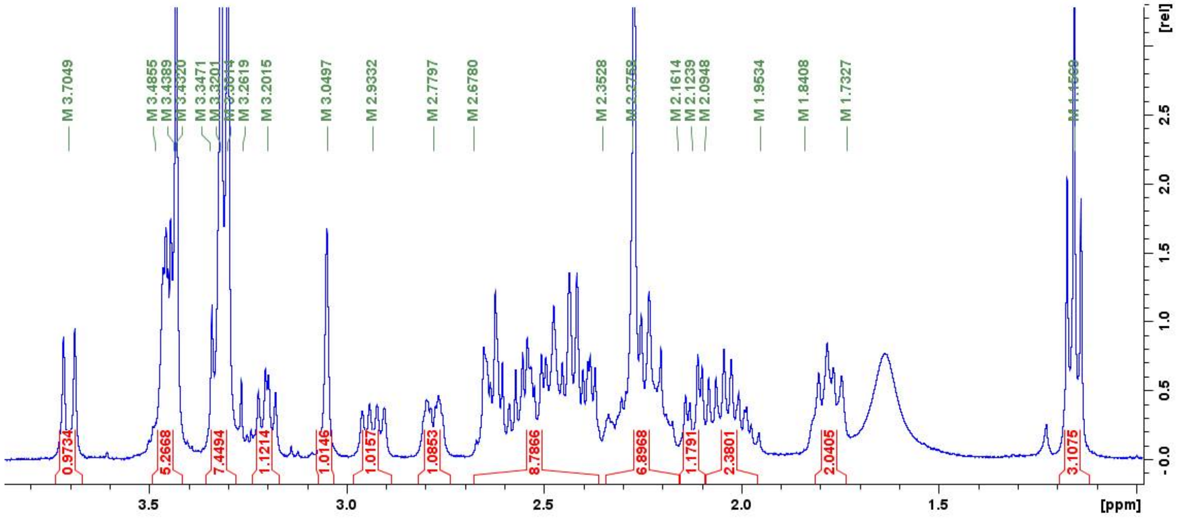

^{13}C NMR (CDCl$_3$, 125 MHz)

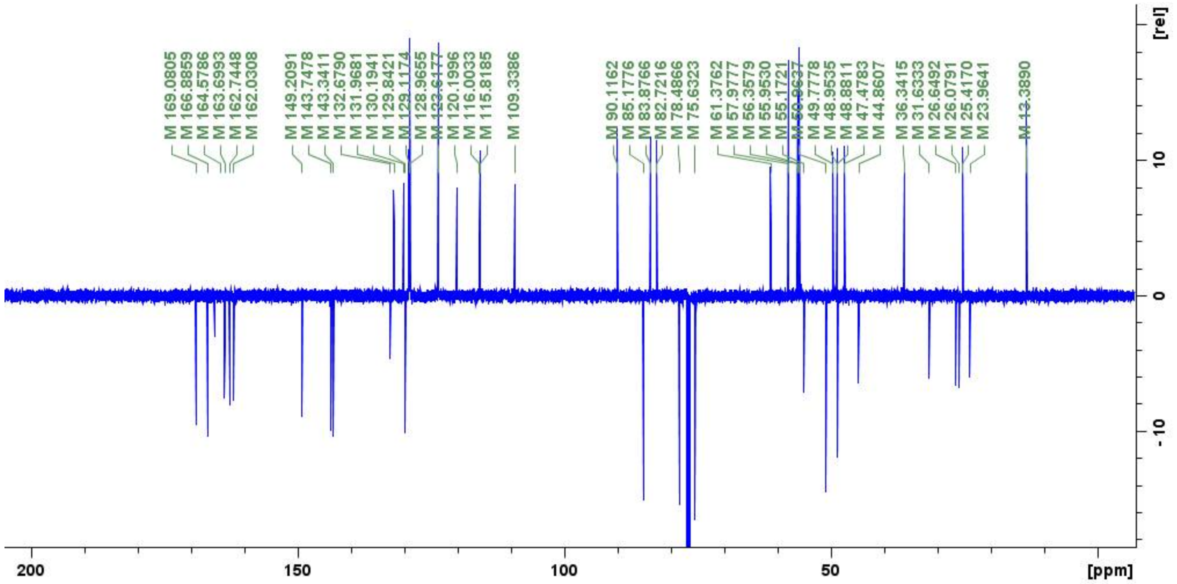

13

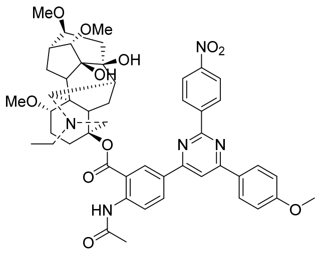

^{1}H NMR (CDCl$_3$, 400 MHz)

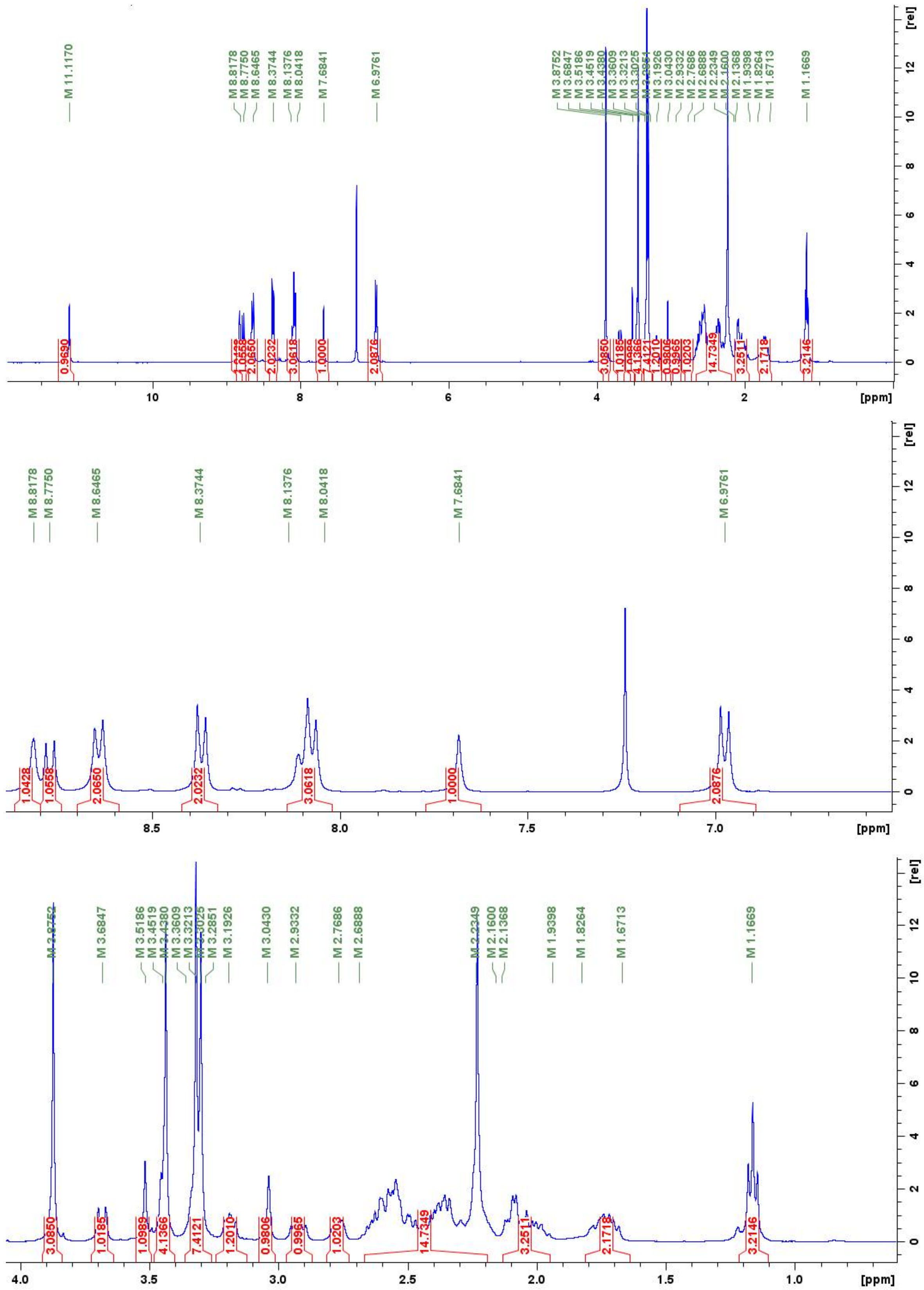

^{13}C NMR (CDCl$_3$, 100 MHz)

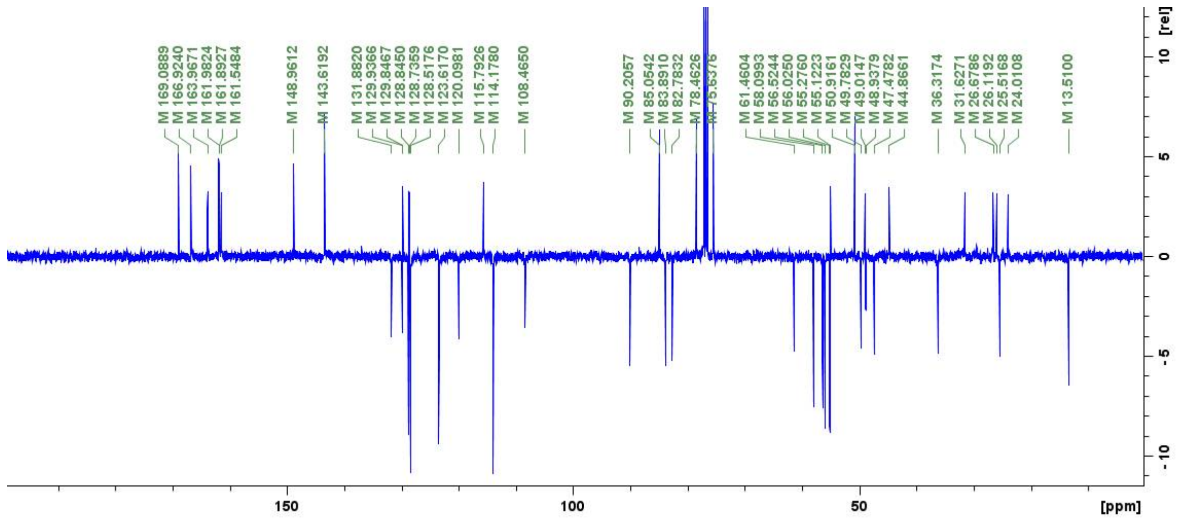

^{13}C NMR (CDCl$_3$, 75 MHz) (bb)

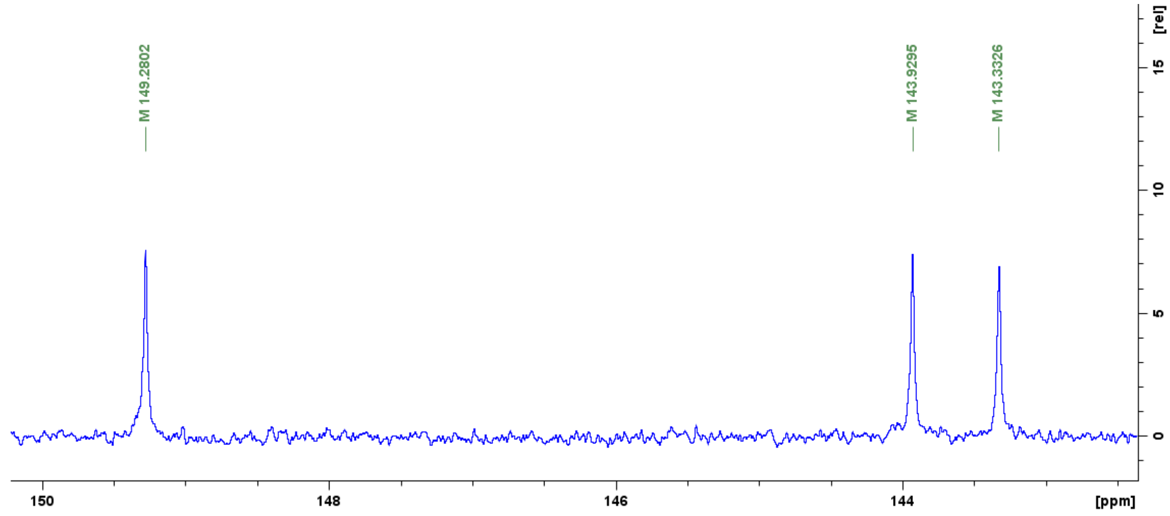

15

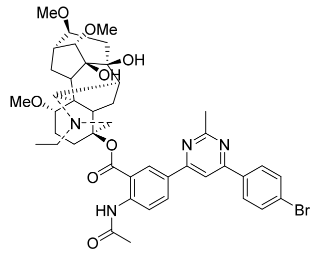

^{1}H NMR (CDCl$_3$, 500 MHz)

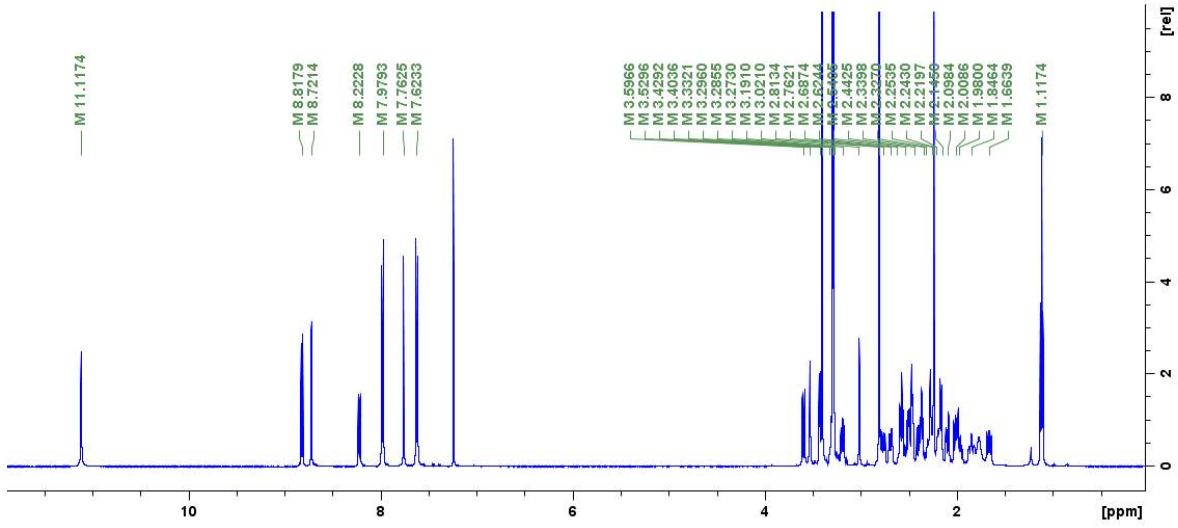

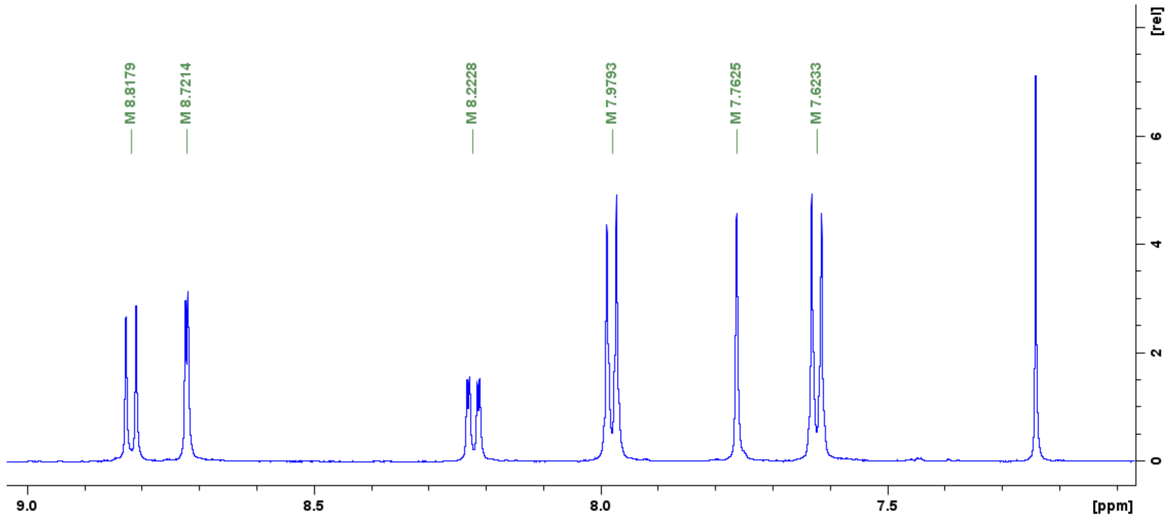

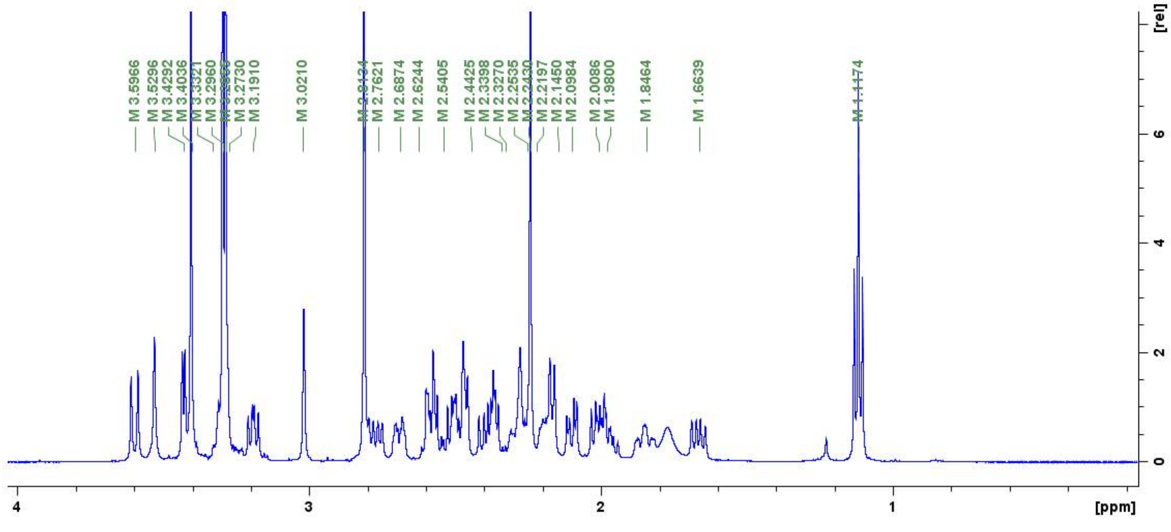

^{13}C NMR ($CDCl_3$, 125 MHz)

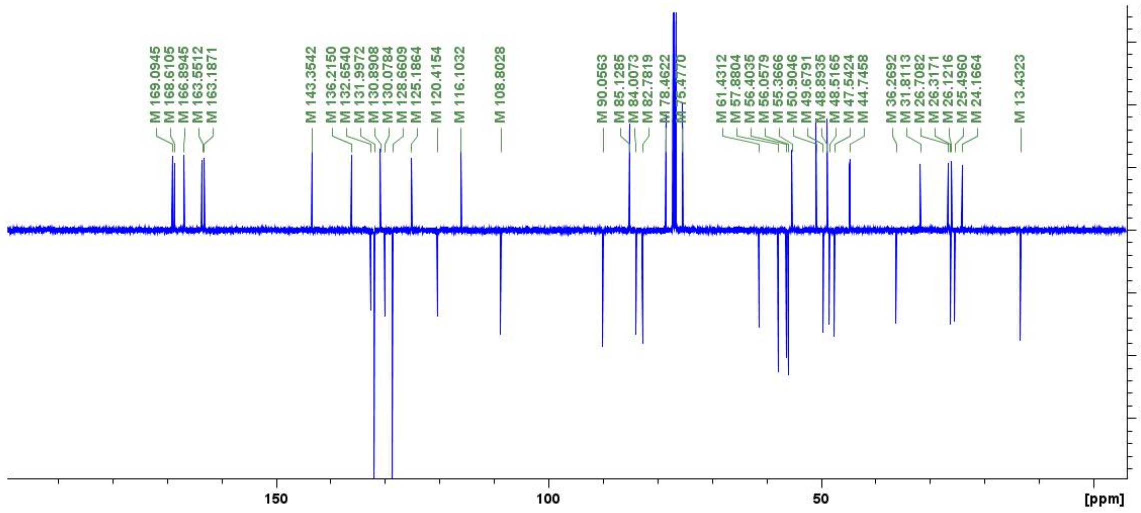

16

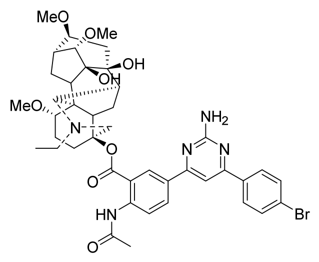

^{1}H NMR (CDCl$_3$, 400 MHz)

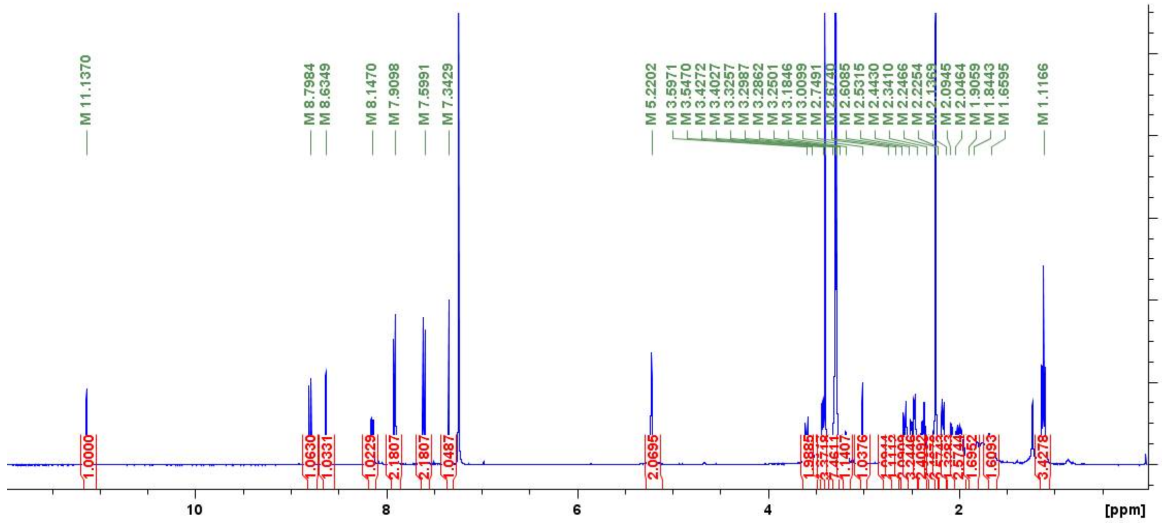

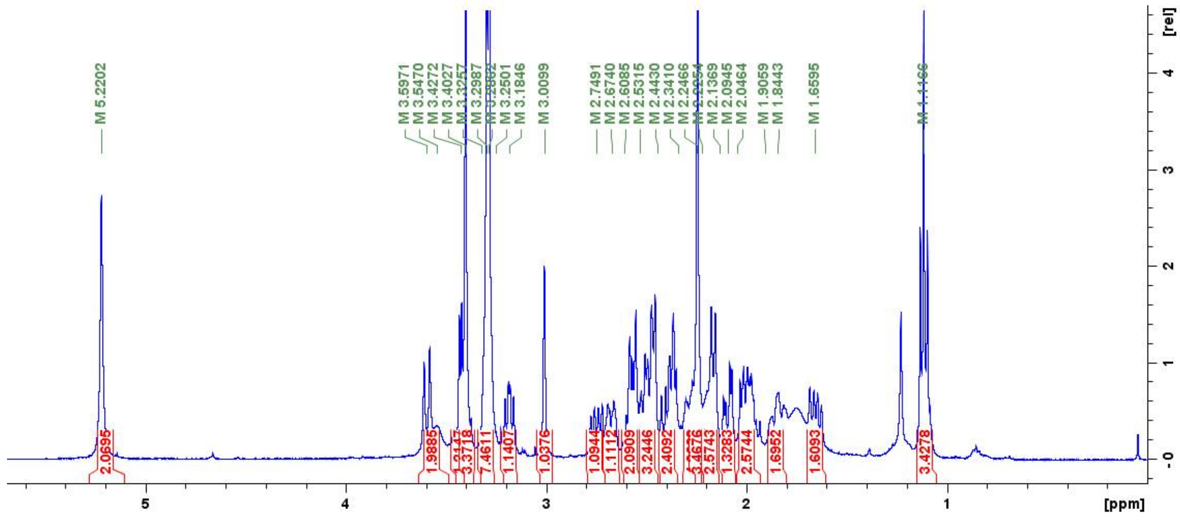

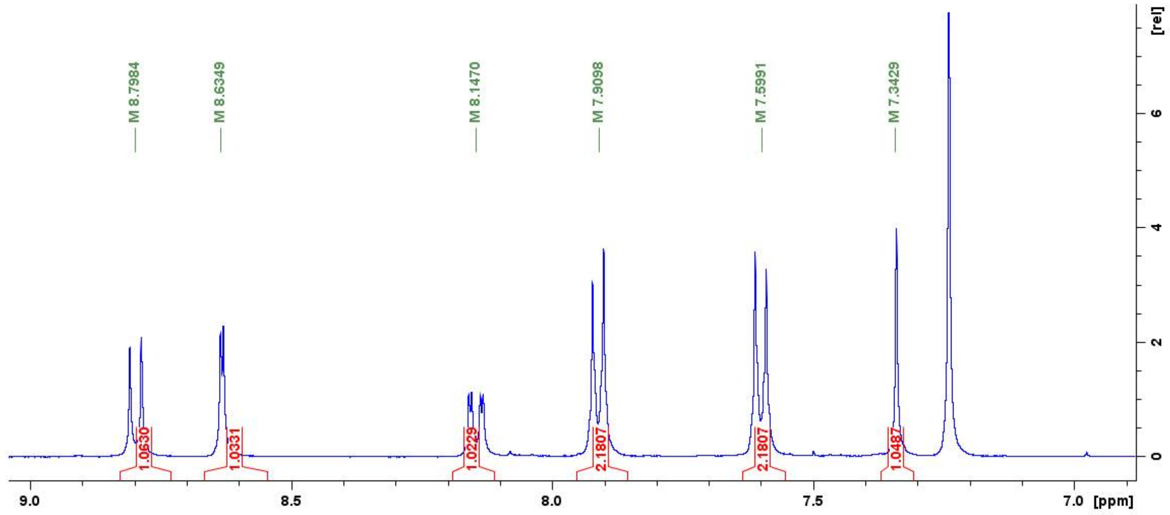

^{13}C NMR ($CDCl_3$, 125 MHz)

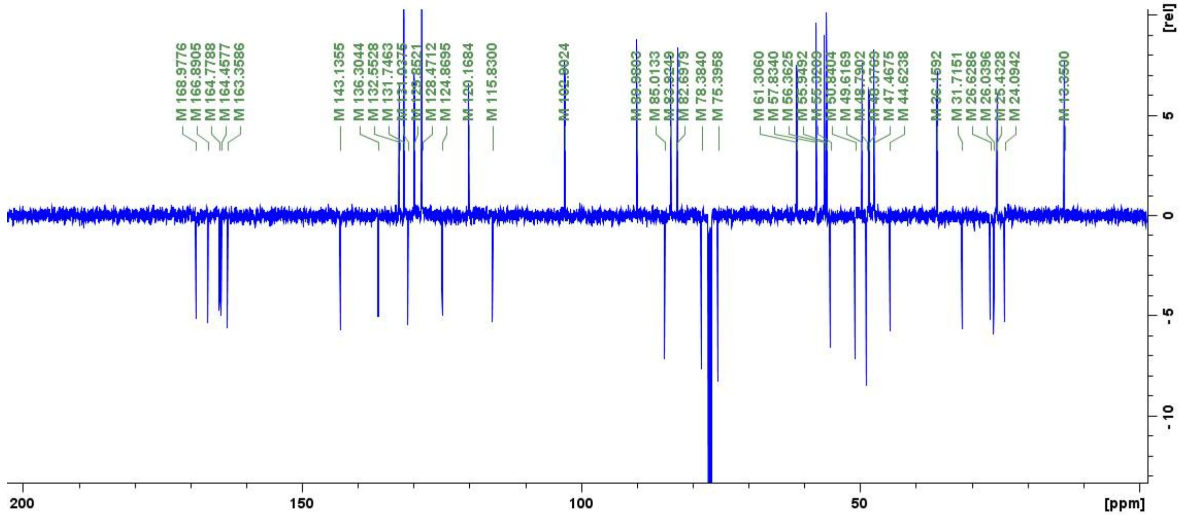

20

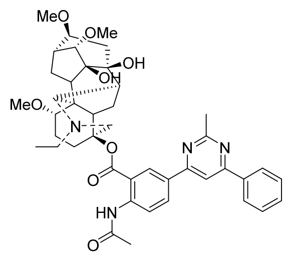

¹H NMR ($CDCl_3$, 500 MHz)

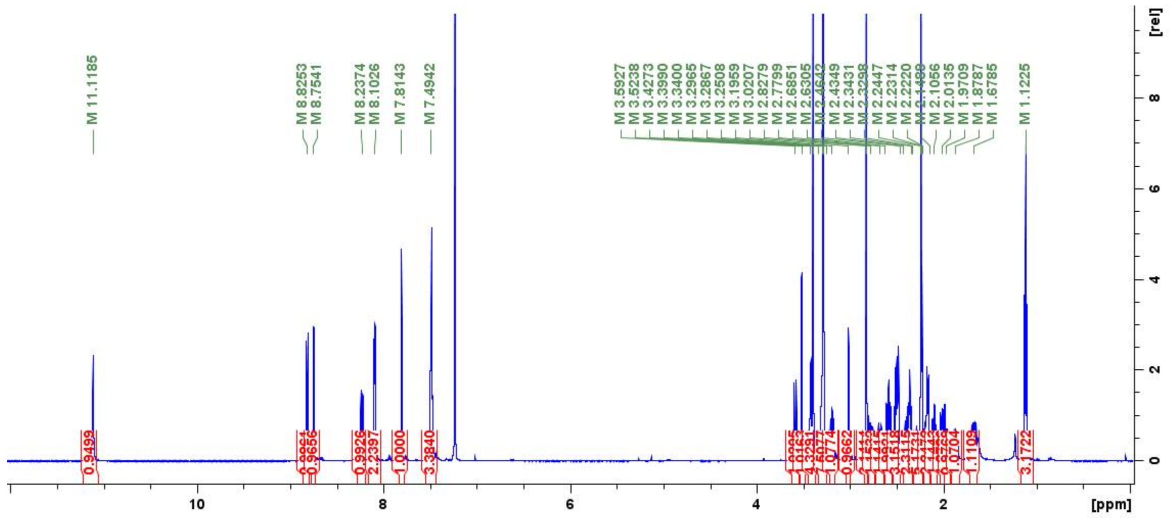

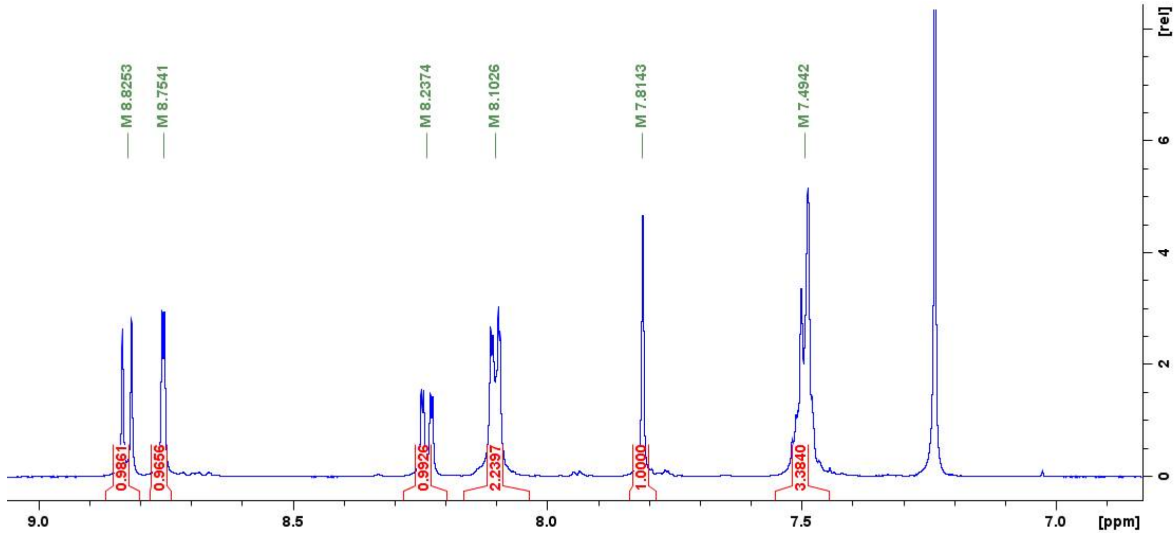

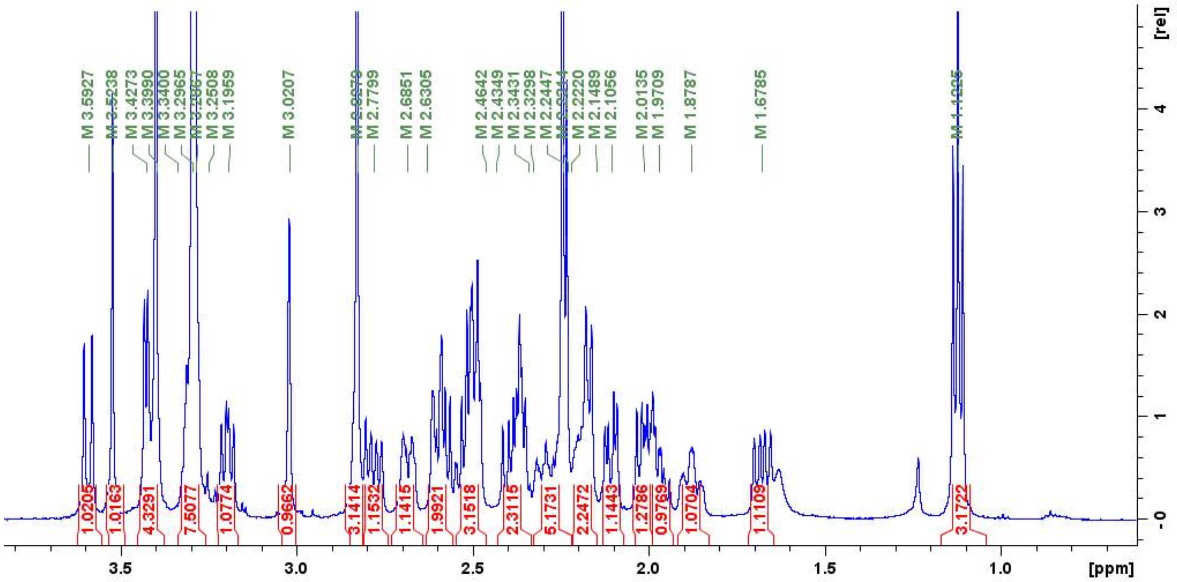

^{13}C NMR (CDCl$_3$, 125 MHz)

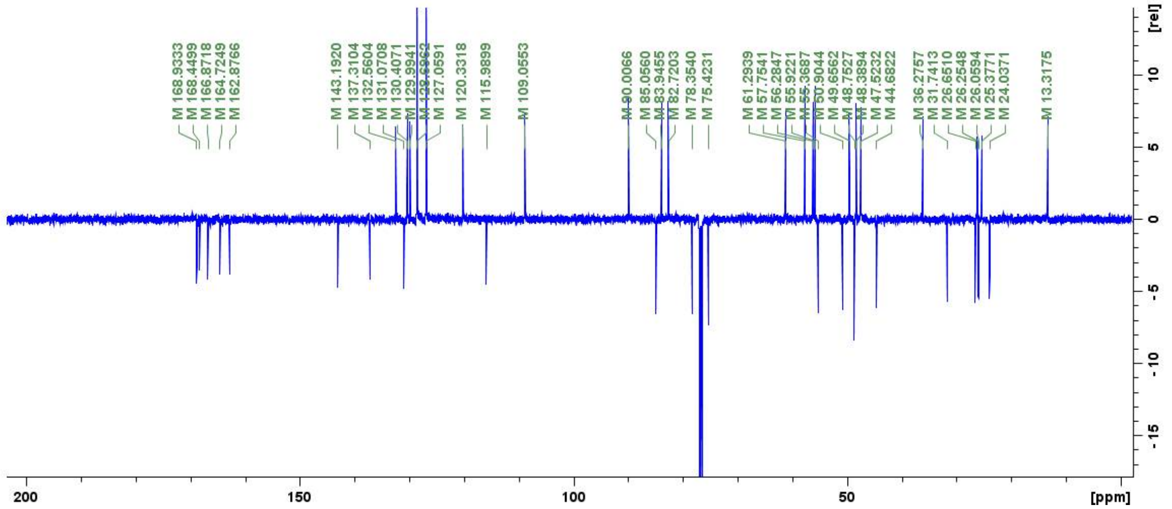

21

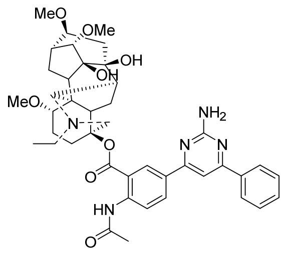

^{1}H NMR (CDCl$_3$, 400 MHz)

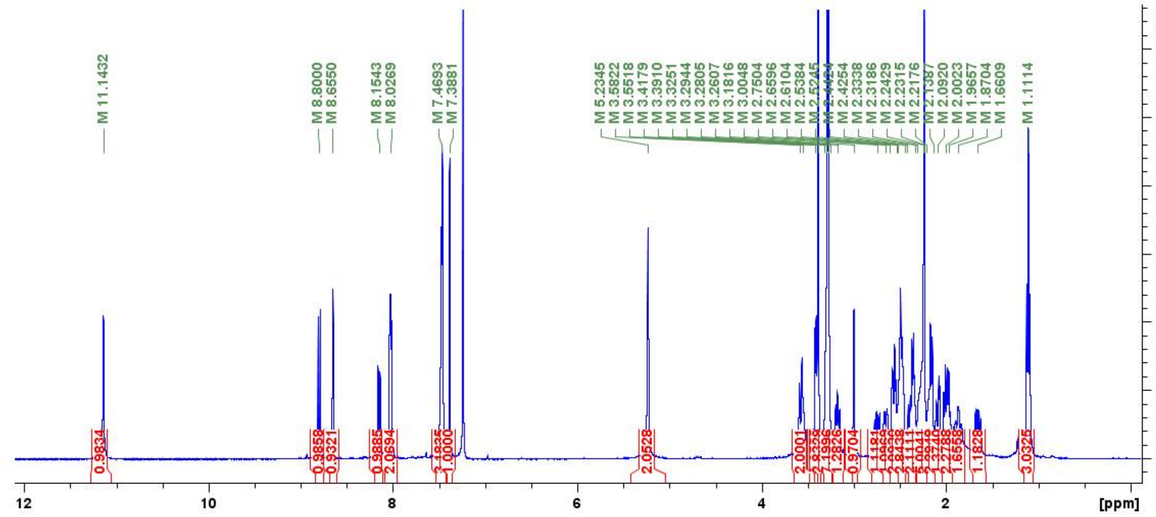

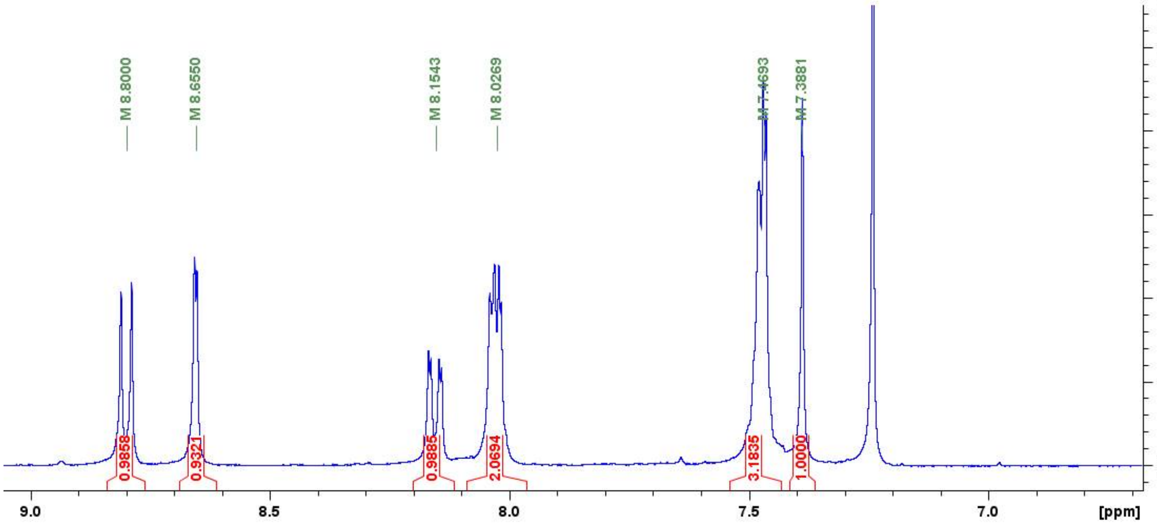

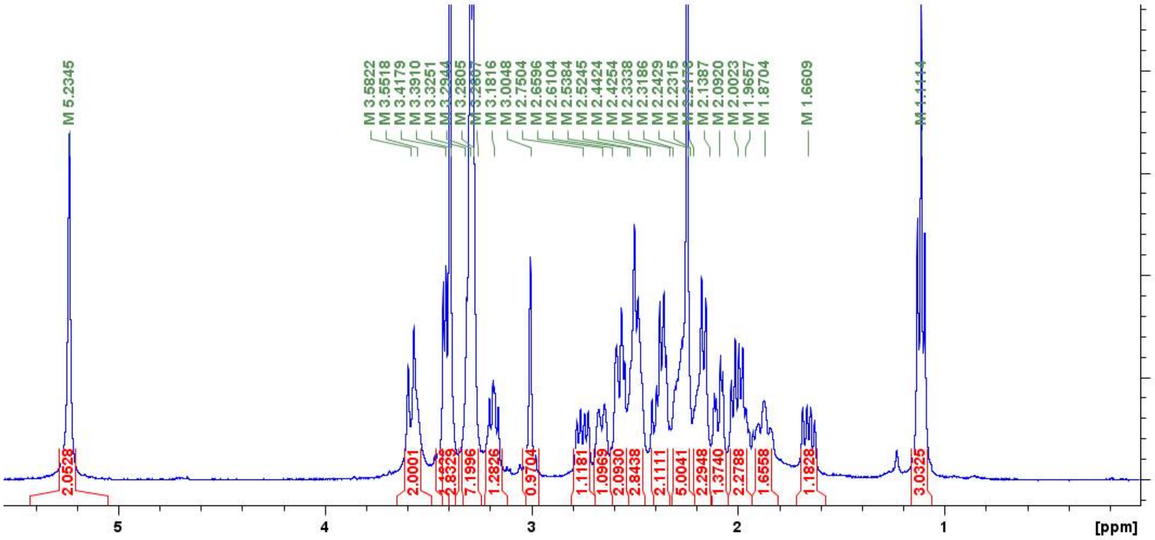

^{13}C NMR ($CDCl_3$, 125 MHz)

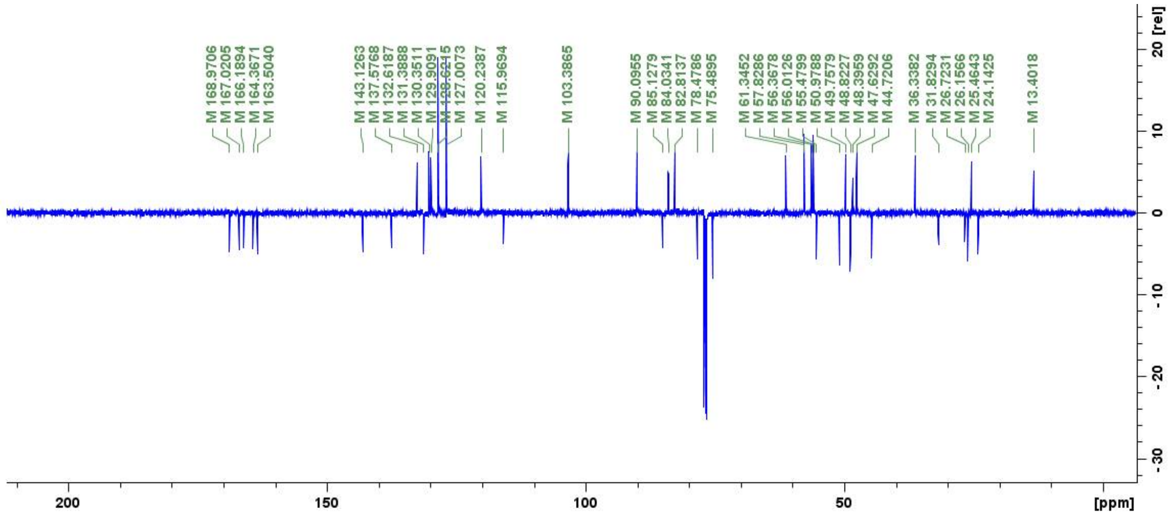

19

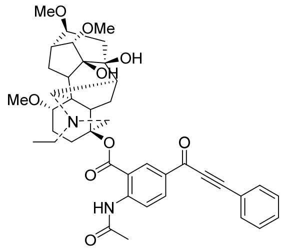

^{1}H NMR ($CDCl_3$, 300 MHz)

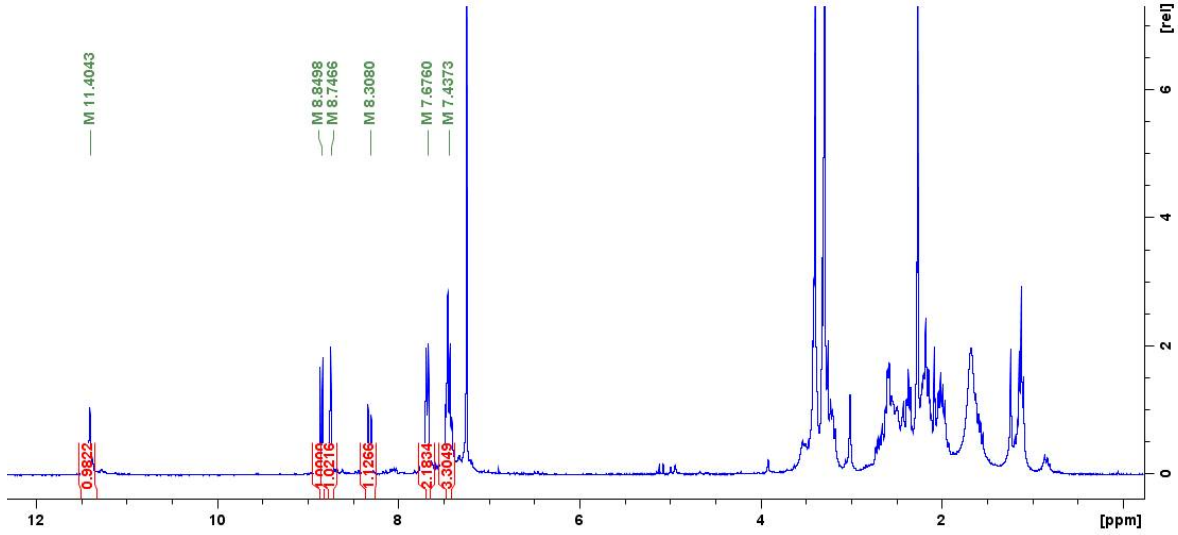

^{13}C NMR ($CDCl_3$, 125 MHz)

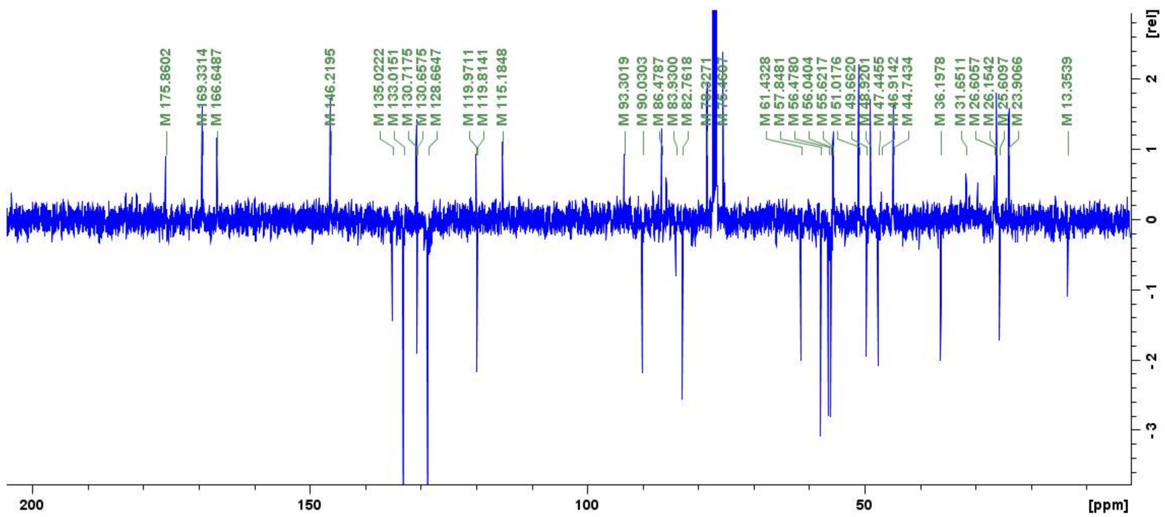
M 175.8602
M 169.3314
M 166.6487
M 146.2195
M 135.0222
M 133.0151
M 130.7175
M 130.6575
M 128.6647
M 119.9711
M 119.8141
M 115.1848
M 93.3019
M 90.0303
M 86.4787
M 83.9300
M 82.7618
M 61.4328
M 57.8481
M 56.4780
M 56.0404
M 55.6217
M 51.0176
M 49.6620
M 47.4455
M 46.9142
M 44.7434
M 36.1978
M 31.6511
M 26.6057
M 25.6097
M 23.9066
M 13.3539
[rel]
[ppm]
